# Excitatory/inhibitory balance emerges as a key factor for RBN performance, overriding attractor dynamics

**DOI:** 10.3389/fncom.2023.1223258

**Published:** 2023-08-09

**Authors:** Emmanuel Calvet, Jean Rouat, Bertrand Reulet

**Affiliations:** ^1^Neurosciences Computationelles et Traitement Intelligent des Signaux (NECOTIS), Faculté de Génie, Génie Électrique et Génie Informatique (GEGI), Université de Sherbrooke, Sherbrooke, QC, Canada; ^2^Département de Physique, Faculté des Sciences, Institut Quantique, Université de Sherbrooke, Sherbrooke, QC, Canada

**Keywords:** reservoir computing, RBN, criticality, attractor, memory, prediction

## Abstract

Reservoir computing provides a time and cost-efficient alternative to traditional learning methods. Critical regimes, known as the “edge of chaos,” have been found to optimize computational performance in binary neural networks. However, little attention has been devoted to studying reservoir-to-reservoir variability when investigating the link between connectivity, dynamics, and performance. As physical reservoir computers become more prevalent, developing a systematic approach to network design is crucial. In this article, we examine Random Boolean Networks (RBNs) and demonstrate that specific distribution parameters can lead to diverse dynamics near critical points. We identify distinct dynamical attractors and quantify their statistics, revealing that most reservoirs possess a dominant attractor. We then evaluate performance in two challenging tasks, memorization and prediction, and find that a positive excitatory balance produces a critical point with higher memory performance. In comparison, a negative inhibitory balance delivers another critical point with better prediction performance. Interestingly, we show that the intrinsic attractor dynamics have little influence on performance in either case.

## 1. Introduction

Reservoir Computing (RC) is a promising field for Machine Learning (ML), as the nonlinear reservoir requires no learning and the readout layer only needs linear regression (Maass et al., 2002; Jaeger and Haas, 2004), reducing time and computational cost (Schrauwen et al., 2007). Furthermore, it has potential for real-world implementations as physical reservoirs and dedicated Neuromorphic chips do not always possess the ability to adapt (Benjamin et al., 2014; Merolla et al., 2014; Tanaka et al., 2019). Around the same time in 2002, two models were developed: the Liquid State Machine (LSM) (Maass et al., 2002) and the Echo State Network (ESN) (Jaeger, 2001) (rectified version). These approaches differ in their neural models, with LSM using time-event-based neurons and ESNs using Artificial Neural Networks (ANN) with continuous activation functions (Jaeger, 2001). The Random Boolean Network (RBN) (Bertschinger and Natschläger, 2004), with binary neurons, is a particularly promising model for LSM and allows for a direct relationship between the reservoir design and its performance in a task (Bertschinger and Natschläger, 2004; Natschläger et al., 2005; Snyder et al., 2012). It is widely used to model and implement reservoirs (Rosin, 2015; Burkow and Tufte, 2016; Echlin et al., 2018; Komkov et al., 2021).

Studies on the RBN have demonstrated the existence of a phase transition in the dynamics of the reservoir for specific connectivity parameters. Close to the critical regime, an increase in performance in solving various tasks has been reported [boolean logic operations (Bertschinger and Natschläger, 2004), bit-parity check (Bertschinger and Natschläger, 2004), prediction of Mackey-Glass time series (Canaday et al., 2018)]. As of now, most studies in the field of RC rely on phase diagrams to exhibit a statistical relationship between connectivity, dynamics, and performance (Bertschinger and Natschläger, 2004; Büsing et al., 2010; Snyder et al., 2012; Krauss et al., 2019a; Metzner and Krauss, 2022). These results have been obtained by considering a limited number of reservoirs [from one (Metzner and Krauss, 2022), to 10 (Bertschinger and Natschläger, 2004), up to 100 (Krauss et al., 2019b)], and with a limited resolution in terms of the control parameter, due to the computational cost of these phase diagrams.

While phase diagrams are essential to comprehend the full range of the computational capabilities these systems can offer, one crucial point is rarely discussed. Since the reservoirs are randomly generated, there might be huge differences between them even though the statistics of their connectivity are the same. Indeed, close to the critical point, reservoir steady-state activities exhibit a wide range of dynamics as discussed by statistical studies (Kinouchi and Copelli, 2006; Del Papa et al., 2017; Krauss et al., 2019b), and attractor classification (Seifter and Reggia, 2015; Bianchi et al., 2016; Krauss et al., 2019a,b; Metzner and Krauss, 2022).

This article aims at studying the variability of reservoir dynamics, performance, and their correlation. We consider randomly generated RBNs with a single control parameter related to the inhibitory/excitatory balance (Krauss et al., 2019a), tuned with high resolution to perform reliable statistical analysis. We study the excitatory/inhibitory balance, attractor dynamics, and performance, and show that the relationship between the three is more complex than previously thought. In line with the work of (Metzner and Krauss, 2022) on ESN, our research reveals that the RBN also possesses two critical points. Depending on whether the balance is in the majority excitatory or inhibitory, we show that reservoirs respectively exhibit optimal performance in either memory or prediction.

The article is organized as follows: in Section 2, we describe the model and prove that it is controlled by the ratio of the standard deviation and mean of the weight distribution (noted σ^⋆^), which we use to perform all subsequent analyses. In Section 3.1, we show that the sign of σ^⋆^ produces two critical regimes. In Section 3.2, we classify the activity of free-running reservoirs into four classes according to their attractor dynamics for these two critical regimes. We show that each reservoir can be associated with its most dominant attractor. In Section 4.1, we evaluate the relationship between connectivity, dominant attractor, and performance in memory and prediction tasks. We then investigate the relationship between the performances of the two tasks, critical regimes, and dominant attractors in Section 4.2. This allows us to derive specific recommendations for simplifying the random generation process of reservoirs. Finally, we discuss our findings in Section 5 and their implications for future works in Section 6.

## 2. Model

**Figure 1:**
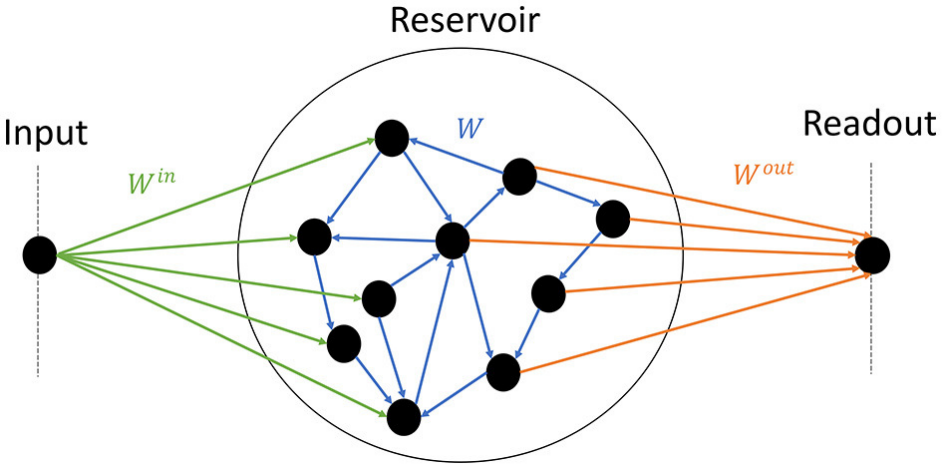
Schematics of the model. The input node **(left)** randomly projects synaptic weights to half of the reservoir **(center)** (green); the reservoir is composed of random recurrent connections (blue); the readout **(right)** receives input from the other half of the reservoir (orange).

The model consists of one input node, the reservoir itself, and an output node (Figure 1). Half of the neurons inside the reservoirs are connected to the input, and the other half to the readout. Thus information between the input and the readout has to pass through the reservoir. The following subsections describe each component and how they are interconnected.

### 2.1. The reservoir

Phase transitions occur *stricto sensu* only in infinite systems, and critical phenomena are easier to observe in large systems (Lavis et al., 2021). As such, we use an RBN model of size *N* = 10, 000 neurons, which is considerable compared to similar studies in the literature (Natschläger et al., 2005; Büsing et al., 2010; Metzner and Krauss, 2022). The binary state *x*_*i*_(*t*)∈{0, 1} of the neuron *i* at the time-step *t* (with *t*∈ℕ), is given by:


(1) $$\begin{array}{l} x_{i}\left(t\right)=\theta \left(u_{i}\left(t\right)+\overset{N}{\underset{j=1}{\sum }}w_{ij}x_{j}\left(t-1\right)\right) \end{array}$$

where θ is the Heaviside step function: θ(*x*) = 1 if *x*> 0 and θ(*x*) = 0 otherwise. Each neuron receives the same number of non-zero connections *K* = 16, in the range of values shown to display sharp phase transitions (Büsing et al., 2010). The non-zero recurrent weights of the reservoir *w*_*ij*_ (blue arrows in Figure 1) are i.i.d. and drawn from the Normal or Gaussian density probability function N(μ,σ). *u*_*i*_(*t*) is the external input of the neuron *i* at times *t*.

### 2.2. Input node

The input layer reduces to one node, receiving the time series *u*(*t*). The input at times *t* of a given neuron *i* is:


(2) $$\begin{array}{l} u_{i}\left(t\right)=w_{i}^{in}u\left(t\right) \end{array}$$

Where the input weight $w_{i}^{in}$, of neuron *i* (green arrows in Figure 1) is drawn from a uniform distribution within [−0.5, 0.5], and half of the weights are set to zero. According to Eq. 1, if the amplitude of the input far exceeds the total contribution of the recurrent weights, then the input mostly controls the dynamics. Our choice of parameters corresponds to an input of zero average and ~0.14 the standard deviation, which is rather low compared to the recurrent weights. We show in part 4.1 that this choice makes the dynamics mostly controlled by the recurrent weights, which is the intended behavior.

### 2.3. Readout

The adaptation mechanism is in the output layer only, which reduces here to one linear node with a sigmoid activation function $f\left(x\right)=\frac{1}{1+e^{-x}}$. As such, the output of the network is given by:


(3) $$\begin{array}{l} y\left(t\right)=f\left(W^{out}\vec{x}+c\right) \end{array}$$

Since all experiments consist in reproducing a unidimensional time series, the output *y* is a scalar as well. The column vector $\vec{x}$ represents the state of the reservoir neurons, while the output weights *W*^*out*^ (orange arrows in Figure 1) are stored in a row vector of size *N*, with half of them set to zero. Lastly, the scalar *c* is the bias. The training is performed with a mean square error (MSE) loss function. Since we had a focus on collecting high-quality data regarding the link between connectivity and performance, we chose the ADAM optimizer (Kingma and Ba, 2015) over the more standard Ridge regression (Burkow and Tufte, 2016) often used in the literature. The implementation is made with the PyTorch library, and parameters α = 0.001, and 4, 000 epochs (see Supplementary material S8.4 for more details).

### 2.4. Connectivity: the control parameter σ^⋆^

To study the reservoir dynamics, one needs the proper definition of a control parameter. Previous work on the RBN often focuses on the average and variance of the recurrent weight matrix (Bertschinger and Natschläger, 2004; Natschläger et al., 2005). In the following, we demonstrate the existence of only one control parameter σ^⋆^ defined by:


(4) $$\begin{array}{l} \sigma^{⋆}=\sigma /\mu ,\;\mu \neq 0 \end{array}$$

Where μ is the mean of the weights and σ their standard deviation. Here we study the reservoir in the absence of external excitation, *u*_*i*_(*t*) = 0 in Eq. (1). Let us consider two reservoirs with the same architecture, the same initial state, and with respective weights matrices *W* and λ*W* with the scalar λ> 0. Since λ> 0, then θ(λ*x*) = θ(*x*), ∀*x*. Thus, according to Eq. (1) for *u*_*i*_(*t*) = 0, the two networks are always in the same state. Thus (λμ, λσ) gives rise to the same time evolution as (μ, σ). The two corresponding reservoirs are totally equivalent. We face two cases depending on μ:

- When μ = 0, choosing λ = 1/σ leads to the conclusion that all reservoirs (0, σ) are strictly equivalent to the reservoir (0, 1). Hence reservoirs with μ = 0 are independent of σ.
- When μ≠0, choosing λ = 1/|μ| leads to the weights of the second reservoir distributed with a mean of ±1 and a standard deviation σ/|μ|. Hence we define the control parameter of the RBN as in Eq. (4).

Equation 4 characterizes the distribution of the weights: the mean is the sign of σ^⋆^, and the standard deviation is its absolute value. Other distribution characterizations directly relate to σ^⋆^. For instance, it is controlling the balance *b* between excitation and inhibition, defined by (Krauss et al., 2019a) as:


(5) $$\begin{array}{l} b=\left(S_{+}-S_{-}\right)/S \end{array}$$

(6) $$\begin{array}{l} S_{\pm }=\frac{S}{2}\left(1\pm b\right) \end{array}$$

With *S* = *S*_+_+*S*_−_ = *KN* the total number of synapses, *S*_−_ the number of inhibitory synapses (*w*_*ij*_ < 0), and *S*_+_ the number of excitatory synapses (*w*_*ij*_> 0). By taking a normal weight distribution, the number of excitatory synapses is given by:


(7) $$S_{+}=S\int_{0}^{+\infty }\frac{1}{\sqrt{\pi }}e^{-{\left(x-\frac{\mu }{\sqrt{2}\sigma }\right)}^{2}}dx$$

**Figure 2:**
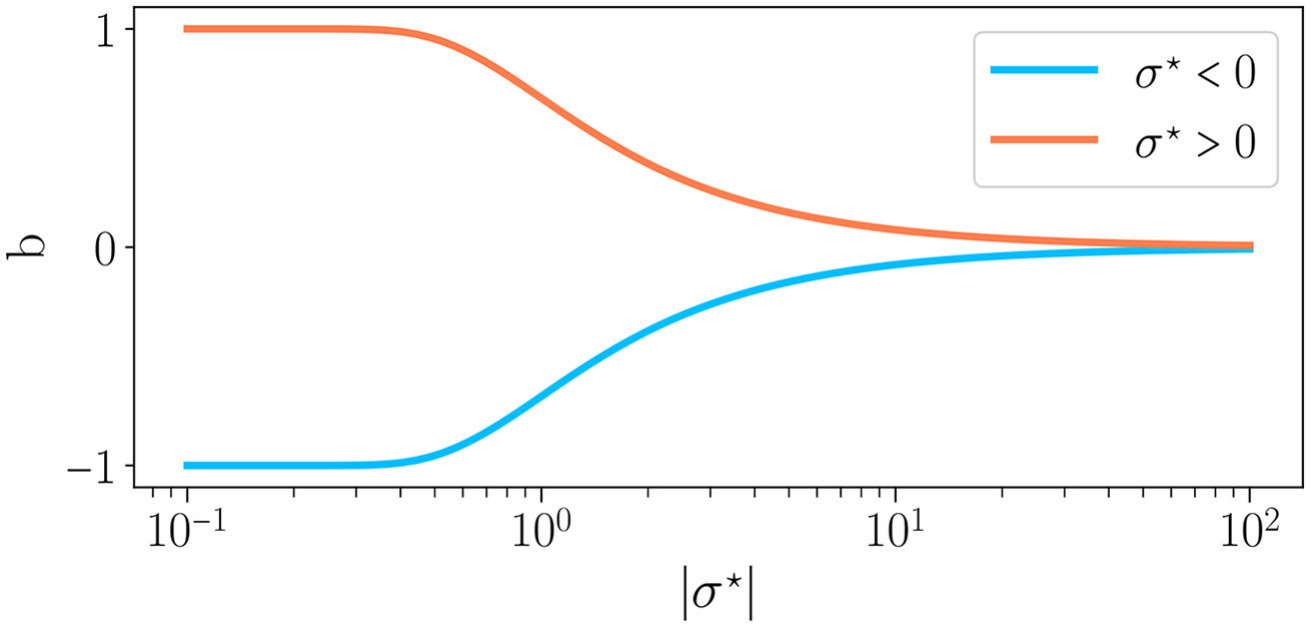
Excitation/inhibition balance *b* as a function of the absolute value of the connectivity parameter σ^⋆^, as defined in Eq. 4, for σ^⋆^ < 0 (—) and σ^⋆^> 0 (—). When the average of weights is positive (σ^⋆^> 0), whereas the reverse is true when the average of weights is negative.

By substituting Eq. (7) in Eq. (6), we find $b=\text{Erf}\left[1/\left(\sqrt{2}\sigma^{⋆}\right)\right]$, with Erf the error function. Thus, by controlling the weight distribution, our control parameter σ^⋆^ drives the excitatory to inhibitory balance and thus the reservoir dynamics, in line with Krauss et al. (2019b) and Metzner and Krauss (2022). Figure 2 shows the relationship between *b* and σ^⋆^. The case μ = 0 corresponds to *b* = 0 (perfect balance between excitation and inhibition) and σ^⋆^ → ∞, for any value of σ. For σ^⋆^ < 0, *b*∈[−1, 0], i.e. there is a majority of inhibitory synapses while for σ^⋆^> 0, *b*∈[0, 1], hence a majority of excitatory synapses.

To finish, the spectral radius ρ(*W*) of the weight matrix *W* is a particularly relevant quantity in the context of the ESN, a continuous version of reservoirs, where the critical point corresponds to a spectral radius of one. However, in the case of discontinuous activation functions, such as the one we have with the RBN, it has been shown that the Echo State Property (ESP) cannot be achieved. The spectral radius alone fails to characterize the dynamics and performance of these reservoirs (Oztuik et al., 2007; Alexandre et al., 2009; Tieck et al., 2018; Balafrej et al., 2022). In Supplementary material S8.1, we explicitly discuss the link between ρ and the mean and variance of the weight matrix and show ρ is of no particular interest in the study of the dynamics.

As a consequence, in the following, we will use σ^⋆^ as the unique control parameter (in the range of values displayed in Supplementary material S8.2).

## 3. Statistics of dynamics at the critical points

The importance of neural networks dynamics in understanding their performances has been widely explored (Bertschinger and Natschläger, 2004; Büsing et al., 2010; Krauss et al., 2019a; Metzner and Krauss, 2022). The purpose of this part is to investigate the relationship between connectivity and dynamics through two statistical analyses:

1. **Activity statistics:** In Section 3.1, we analyze the statistics of the neural activity as a function of the control parameter σ^⋆^. We demonstrate the existence of two critical points and characterize them.
2. **Reservoirs attractors:** In Section 3.2, we classify the steady state time evolution of the network activity into four distinct attractors and study the influence of the initial state and random weight generation. We show that reservoirs possess a dominant attractor independent of initial conditions.

### 3.1. Statistics of the activity of free-evolving reservoirs

#### 3.1.1. Methodology

The first experiment is a free evolution of reservoirs in the absence of input, i.e. *u*_*i*_ = 0. We define the network activity as $A\left(t\right)=\underset{i}{\sum }x_{i}\left(t\right)/N$, with *N* the number of neurons in the reservoir, and *A*∈[0, 1]. *A* is also the proportion of excited neurons: *A* = 0 if the network is extinguished (*x*_*i*_ = 0 ∀*i*), *A* = 1 if the network is saturated (*x*_*i*_ = 1 ∀*i*). At the initial state, we randomly force 20% of neurons to an up state (*x*_*i*_ = 1), i.e. *A*(*t* = 0) = 0.2. After a transient regime of 1, 000 time steps, the reservoir reaches a steady state where we perform statistics. In the following, *A* will refer to the activity measured in that steady state (see Supplementary material S9 for a more formal definition). For each value of σ^⋆^, we perform statistics on 100 randomly generated reservoirs (see Supplementary material S7.1 for more details on the experiment).

#### 3.1.2. Analysis

In the following, a bar over a variable $\bar{\left(.\right)}$ represents an average over time for a given reservoir, while the brackets 〈.〉 represents an average over different randomly generated reservoirs. In the first analysis, we calculate the time-average steady activity Ā for a given reservoir and its time-variance $\bar{\delta A^{2}}$, where we define δ*A* = *A*−Ā. We average these quantities over the reservoirs to give 〈Ā〉 and $\langle \bar{\delta A^{2}}\rangle$ for each value of σ^⋆^. Next, we evaluate the average and variance over reservoirs of the binary entropy *H*_*b*_, or BiEntropy (Croll, 2014) of the time-dependent activity. Compared to the Shanon entropy, the advantage of this metric is that it can discriminate ordered from disordered strings of binary digits. It has been used in machine learning (Mamun et al., 2016; Zhou and Zeng, 2022), but to our knowledge, this is the first time in reservoir computing. The binary entropy varies between 0 for fully ordered bit-streams and 1 for fully disordered ones. We compute the BiEntropy of the binarized time dependence of the steady activity for each reservoir (for the exact definition of all the metrics, see Supplementary material S10).

#### 3.1.3. Results

**Figure 3:**
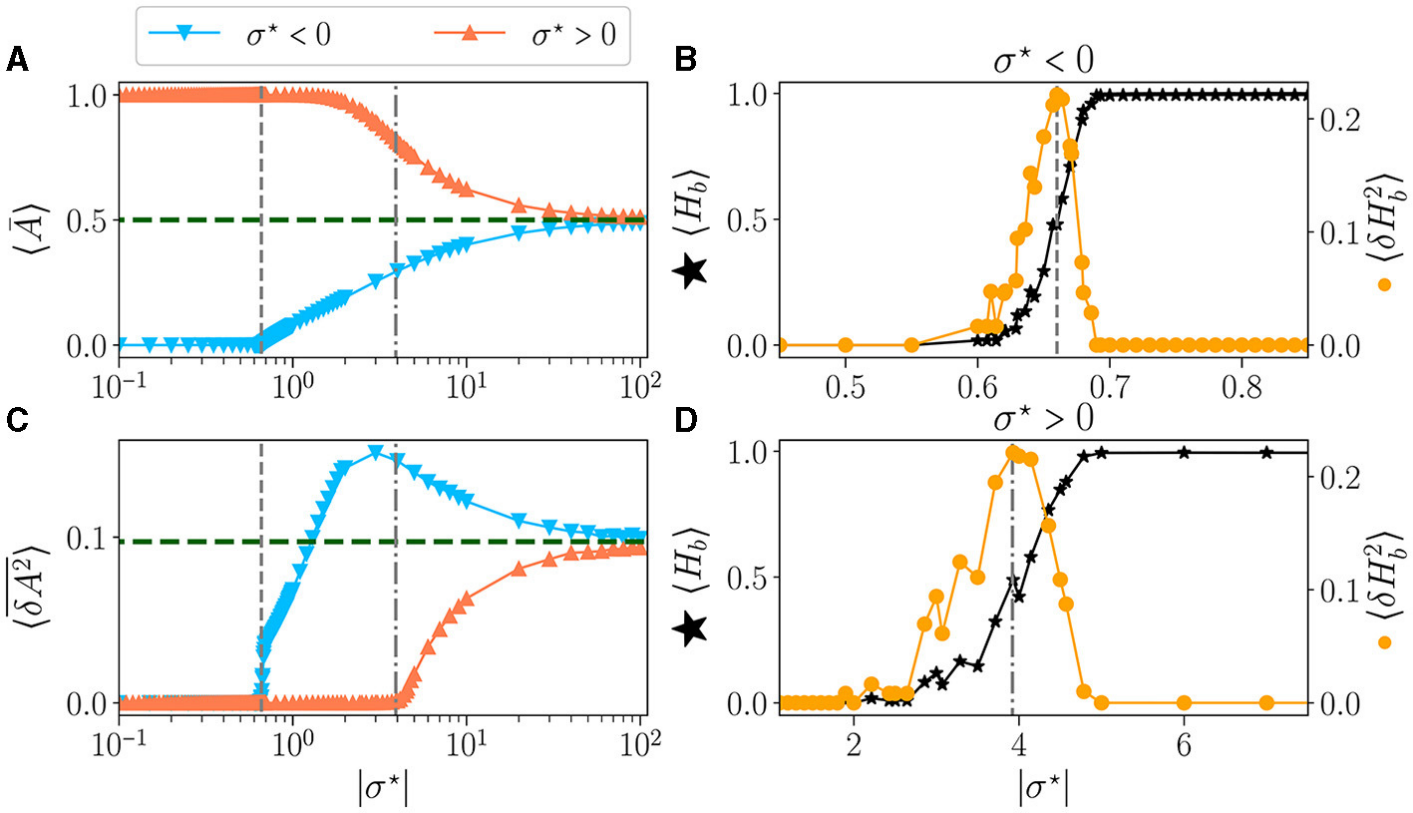
Statistics of the activity of free running reservoirs in the steady state as a function of |σ^⋆^|. Each dot represents the statistics over 100 reservoirs ran once. Average over reservoirs of time average activity 〈Ā〉 **(A)**, and average over reservoir of time variance $\langle \bar{\delta A^{2}}\rangle$ **(C)**, for σ^⋆^ < 0 (▾) and σ^⋆^> 0 (▴). In all plots, the gray vertical lines represent the critical values of the control parameter for $\sigma_{c}^{⋆}<0$ (*---*) and $\sigma_{c}^{⋆}>0$ (-.-.). **(B**, **D)** Zoom on the region of interest close to the critical points: average over reservoirs of BiEntropy 〈*H*_*b*_〉 (⋆, left scale) and BiEntropy variance $\langle \delta H_{b}^{2}\rangle$ (•, right scale), for σ^⋆^ < 0 **(B)** and σ^⋆^> 0 **(D)**.

The time-average activity 〈Ā〉 as a function of σ^⋆^ is shown in Figure 3A, for both signs of σ^⋆^ (Figure 2). The green dashed line represents the value obtained for μ = 0, i.e. σ → ∞. The perfect balance in excitation (*b* = 0) results in half of the neurons being activated 〈Ā〉 = 0.5. The variance $\langle \bar{\delta A^{2}}\rangle$ vs. σ^⋆^ is shown in Figure 3C. For the lowest values of |σ^⋆^|, the reservoirs are frozen (zero variance) either extinguished (for σ^⋆^ < 0) or saturated (σ^⋆^> 0). This corresponds to reservoirs being respectively purely inhibitory (*b* = −1) or excitatory (*b* = 1). Already at the level of the statistics of the activity, there is a clear difference between both signs of σ^⋆^: for σ^⋆^ < 0 there is a threshold in σ^⋆^ (vertical dashed line at σ^⋆^~−0.7) above which the average activity and its variance rise abruptly and simultaneously. In contrast, for σ^⋆^> 0, there is a wide region where no dynamic is detected (zero variance), yet the network is not saturated but its activity decays continuously. The variance starts rising at σ^⋆^> 4 (vertical dash-dotted line).

The average BiEntropy 〈*H*_*b*_〉 vs. |σ^⋆^| is plotted in Figure 3B for σ^⋆^ < 0 and Figure 3D for σ^⋆^> 0 (left scale, black stars on both plots). These two plots zoom in the vicinity of the phase transition, as statistics are stationary elsewhere. There is a continuous transition between a fully ordered phase (〈*H*_*b*_〉 = 0) and a fully disordered one (〈*H*_*b*_〉 = 1). Since the BiEntropy is a measure of order, these results suggest that the transition we observe is related to the apparition of chaos in the reservoir above a critical value of σ^⋆^ (Lewin and Bak, 1993; Bertschinger and Natschläger, 2004; Seifter and Reggia, 2015; Kuśmierz et al., 2020). The variance of the BiEntropy $\langle \delta H_{b}^{2}\rangle$ is shown in Figure 3B for σ^⋆^ < 0 and Figure 3D for σ^⋆^> 0 (right scale, orange circles on both plots). It is zero when either in the ordered or disordered phase and spikes at the transition. Its maximum coincides with 〈*H*_*b*_〉≃0.5: the variance of BiEntropy captures the edge of chaos as a balance between order and disorder. More striking, it also coincides with the position at which the variance of the activity rises (vertical dashed lines). The peak of $\langle \delta H_{b}^{2}\rangle$ thus provides a clear definition of the position of two critical points: $\sigma_{c}^{⋆}≃-0.66$ and $\sigma_{c}^{⋆}≃4.0$, which correspond respectively to critical balances *b*_*c*_≃−0.87 (94% of inhibitory synapses) and *b*_*c*_≃0.19 (60% of excitatory synapses). Moreover, the transition between order and disorder is much wider for σ^⋆^> 0. This asymmetry between both signs of σ^⋆^ is a property of our model since θ(−*x*)≠±θ(*x*) in Eq.(1). From now on, we will refer to the *critical points* as the point where the maximum of BiEntropy variance is obtained, and we will define the *critical regions* as the regions with $\langle \delta H_{b}^{2}\rangle \neq 0$.

#### 3.1.4. Discussion

Similar to (Krauss et al., 2019b) and (Metzner and Krauss, 2022), the existence of phases separated by critical points as a parameter is varied is reminiscent of the phase diagrams drawn in thermodynamics. If we associate the state of a neuron, 0 or 1, to the state of an Ising spin, either down or up, then 〈Ā〉 corresponds to the average magnetization per spin of the network and $\langle \bar{\delta A^{2}}\rangle$ to the variance of its fluctuations, i.e., magnetization noise. At equilibrium, it is proportional to the magnetic susceptibility according to the fluctuation-dissipation theorem (Callen and Welton, 1951). The total magnetization plays the role of an order parameter, and the transition order is obtained by considering discontinuities, as a function of temperature, of the order parameter and its derivatives with respect to the external field (Landau and Lifshitz, 1980). Here we observe that the average activity is always continuous as a function of σ^⋆^. At the same time, $\langle \bar{\delta A^{2}}\rangle$ is continuous for σ^⋆^> 0 but shows a discontinuity at the critical point for σ^⋆^ < 0. This strongly suggests that the two “phase transitions” are of a different type.

### 3.2. Dominant attractor of reservoirs

In the previous section, we considered the *average* behavior of reservoirs: for a given value of σ^⋆^ we averaged over many realizations of the distribution of synaptic weights. However, from a practical point of view, one wants to use one network to work with different inputs. This raises two questions: that of the reservoir-to-reservoir variability (do all reservoirs behave similarly?) and that of the sensitivity of a given reservoir to initial conditions. We address these questions in this section.

#### 3.2.1. Methodology

We submitted our reservoirs again to a free evolution without input [*u*_*i*_(*t*) = 0]. For each value of σ^⋆^, we created 100 reservoirs with randomly tossed weight matrices. Each reservoir is run 100 times, with a different random initial state, of activity *A*(*t* = 0) = 0.2 (for more details, see *Statistics of reservoirs* in Supplementary material S7).

#### 3.2.2. Analysis

We classify the attractor obtained in the steady-state activities, as proposed in (Krauss et al., 2019a). We categorize the activity signals into one of the four types of attractors (see Supplementary material S11 for a grounded justification of each category):

- *Extinguished* activity: The steady-state activity *A*(*t*) is always zero. This means that the initial activity died during the transient phase and that the reservoir could not propagate it further in time. For simplicity, we will sometime refer to it as *dead* attractor.
- *Fixed* point attractor: The steady-state reservoir is active [*A*(*t*)≠0], but the activity is independent of time [δ*A*(*t*) = 0]. This includes the *saturated* states *A*(*t*) = 1 of Seifter and Reggia (2015).
- *Cyclic* attractor: *A*(*t*) is periodic with a periodicity larger than one time-step.
- *Irregular* attractor: *A*(*t*) is neither constant nor periodic within the duration of the simulation. Note that since the RBN is finite, discrete, and deterministic, given enough time, any sequence of states should eventually repeat, taking at most 2^*N*^ time steps.

We determine the attractor obtained at the steady state for each reservoir and initial condition. We then compute the distribution of attractors for each value of σ^⋆^ obtained overall the initial conditions of all reservoirs. The statistics are thus computed on 10,000 steady activities for each σ^⋆^.

#### 3.2.3. Results:

**Figure 4:**
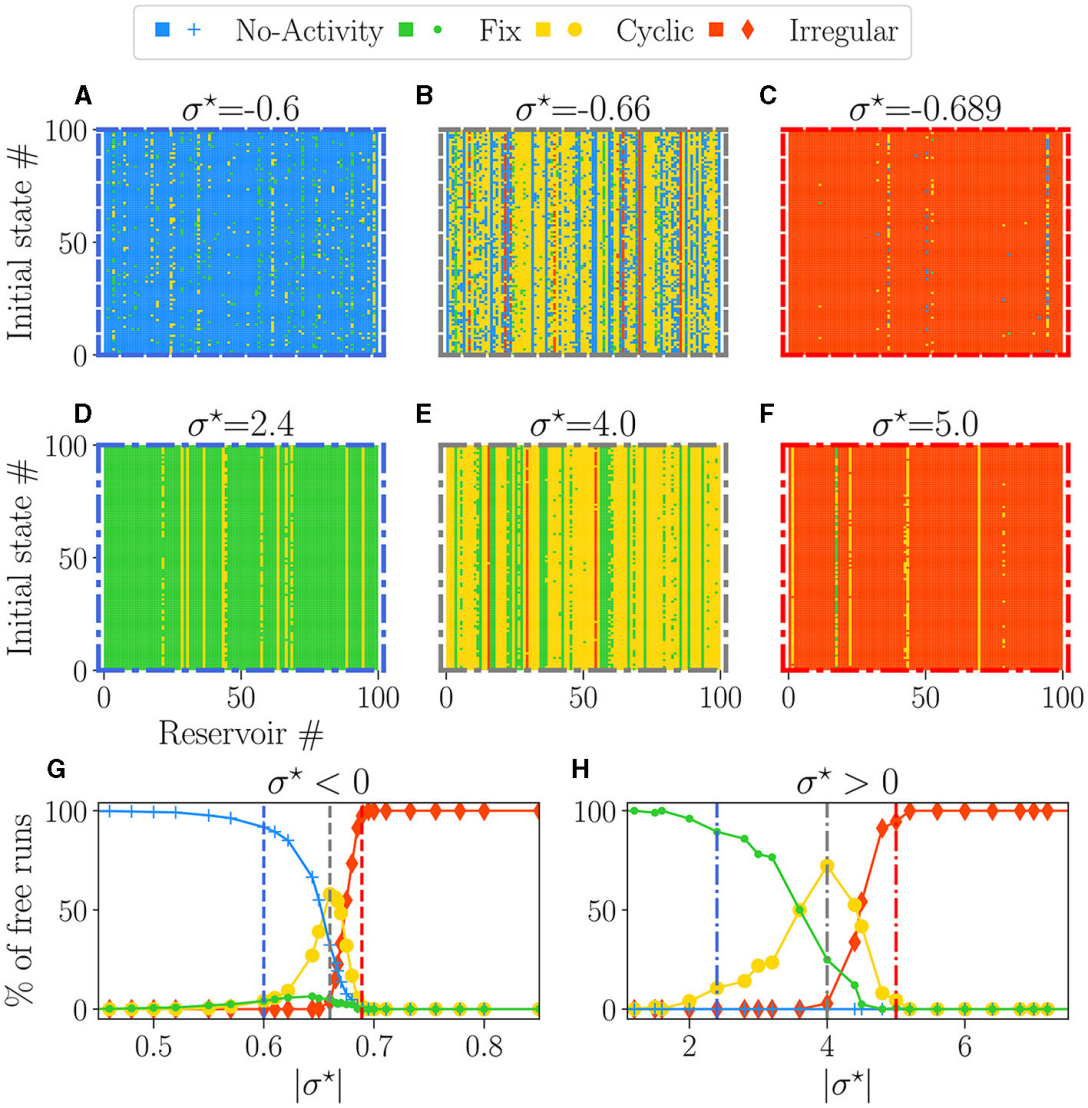
The attractor landscape of reservoirs: for σ^⋆^ < 0 **(A–C**, **G)**, and for σ^⋆^> 0 **(D–F**, **H)**. The influence of initial conditions for specific values of σ^⋆^: **(A)** σ^⋆^ = −0.6, **(B)** σ^⋆^ = −0.66, **(C)** σ^⋆^ = −0.689, **(D)** σ^⋆^ = 2.4, **(E)** σ^⋆^ = 4.0, **(F)** σ^⋆^ = 5.0. In each plot, the vertical axis represents different numbers (#) of initial random states of free-running reservoirs. The horizontal axis represents different numbers (#) of reservoirs with various initial weight tossing, randomly generated with distinct seeds. Pixels of colors represent the attractor obtained at the steady state, with the same colors as **(G, H)**. **(G, H)** Percentage of steady-state activities belonging to each category of attractors: no-activity (+), fix (•), cyclic (•), and irregular (♦). Each dot represents the statistics over 100 reservoirs ran 100 times, hence 1, 000 runs. In each row **(A–C, E–G)**, the colored dashed boxes surrounding the plots correspond to the values of σ^⋆^, indicated as vertical lines in plots **(G, H)**.

Figures 4A–F provide examples of attractors, encoded in the colors, for each reservoir (x-axis) and each initial condition (y-axis) for different values of σ^⋆^. The left column (blue-bordered boxes) corresponds to values below the critical point (vertical blue lines on Figures 4G, H), the center column (gray-bordered boxes) to values at the critical point (vertical gray lines), and the right column (red-bordered boxes) to values above the critical points (vertical red lines). The upper row displays negative σ^⋆^ values, while the lower row features positive σ^⋆^ values.

Away from the critical point, a dominant color is observed, meaning that reservoirs exhibit a dominant attractor. Steady activities are predominantly extinguished and fixed on the left side of the critical point (Figures 4A, D) and irregular at the right (Figures 4C, F). Close to the critical points (Figures 4B, E), there is an increase in the diversity of attractors, as previously observed (Karimipanah et al., 2017).

Figures 4G, H show the statistical distribution of all obtained attractors vs. |σ^⋆^|. As expected from the previous analysis, there is no attractor diversity on the far left and right of the plots, as we obtain one primary attractor. Dead (blue line) or fixed (green line) attractors are found for low values of |σ^⋆^|, and their proportion decays slowly across the transition. Within the critical region coexist all attractors in various proportions. Chaotic attractors start to appear precisely at the transition (vertical gray lines), while the domain where cyclic attractors exist coincides with the critical region of nonzero BiEntropy variance (Figures 3B, D). The point at which cyclic attractors are most present is also precisely $\sigma_{c}^{⋆}$. These results corroborate what we inferred in the previous section: on the disordered phase $\left|\sigma^{⋆}|>|\sigma_{c}^{⋆}\right|$, attractors are irregular, while the ordered phase is characterized by fixed or dead attractors. We note an asymmetry between both sides of the transition: irregular attractors appear only in the disordered phase. From the point of view of the attractors, both signs of σ^⋆^ lead to similar behaviors, except again, that the transition region is much wider for σ^⋆^> 0.

#### 3.2.4. Discussion

Our results suggest that the critical points enhance sensitivity to the initial states and configuration of the weights, explaining the reservoir-to-reservoir variance and increase in dynamic diversity. Reservoirs around the negative $\sigma_{c}^{⋆}$ (Figure 4B) possessed a distribution of attractors with far more variety than the one with positive $\sigma_{c}^{⋆}$ (Figure 4E), further reinforcing the idea that the sign of σ^⋆^ produces two distinct types of critical regimes. We quantified this in the Supplementary material S12 by computing the entropy of reservoir attractor distributions plotted in Supplementary Figure 9. We interpret that result by suggesting that inhibition might be a key factor for enhancing dynamic diversity.

For the purpose of reservoir design, our findings suggest that with both critical points, most reservoirs possess an attractor obtained predominantly in most trials, independent of the initial state. The statistics of dominant reservoir attractors are presented in Supplementary Figure 9, and found to be similar to the one in Figure 3. The presence of vertical color lines in Figures 4A–F means that, in most cases, the behavior of the reservoirs does not depend on the initial state, even in the critical region (this is more thoroughly shown in the Supplementary material S12). As a consequence, a dominant attractor can be associated with each reservoir, irrespective of the initial condition.

## 4. What drives performances

In this section, we examine whether there is a relationship between reservoir dynamics in the absence of input, as explored in the previous section, and its ability to perform two demanding tasks: memory and prediction. This is done in two steps:

- Connectivity, attractor, and performance: In Section 4.1, we analyze the performance obtained separately in each task, depending on the control parameter, for each dominant attractor category. We show that the key factor driving performance is the excitatory/inhibitory balance.
- Attractor and cross-task performance: In section 4.2, we analyze all reservoir performances independently of the control parameter. For each reservoir, we study the relationship between the performance obtained in each task and their dominant attractor. This allows us to deduce how to generate a reservoir for the best general purpose.

From now on, and for ease of notation, a reservoir with a dominant attractor obtained during free evolution (defined in previous Section 3.2) will be referred to as either: a *extinguished, fix, cyclic*, or *irregular* reservoir (e.g., an *extinguished reservoir* refers to a reservoir with an extinguished dominant attractor).

### 4.1. Performance in memory and prediction tasks

Close to the critical points, we obtained various dominant attractors for a single value of σ^⋆^. This raises an important question regarding the relationship between the dominant attractor of a reservoir and its performance. Specifically, it is worth investigating whether the dominant attractor influences the reservoir's performance. If this is the case, grouping attractor categories by discrete performance levels may be possible based on a single value of σ^⋆^.

#### 4.1.1. Methodolody

We evaluate the performance of the networks to execute two fundamental tasks: *memory* and *prediction*. Each reservoir receives an input *u*(*t*), and the readout target is *T*(*t*) = *u*(*t*+δ), equal to the input shifted in time by δ time steps. δ < 0 corresponds to a memory task, and δ> 0 to a prediction task. For each value of σ^⋆^, we use 100 reservoirs, and each reservoir is run five times, with a different random tossing of the input weight matrix (more detail on the training procedure in Supplementary material S8.4).

**Figure 5:**
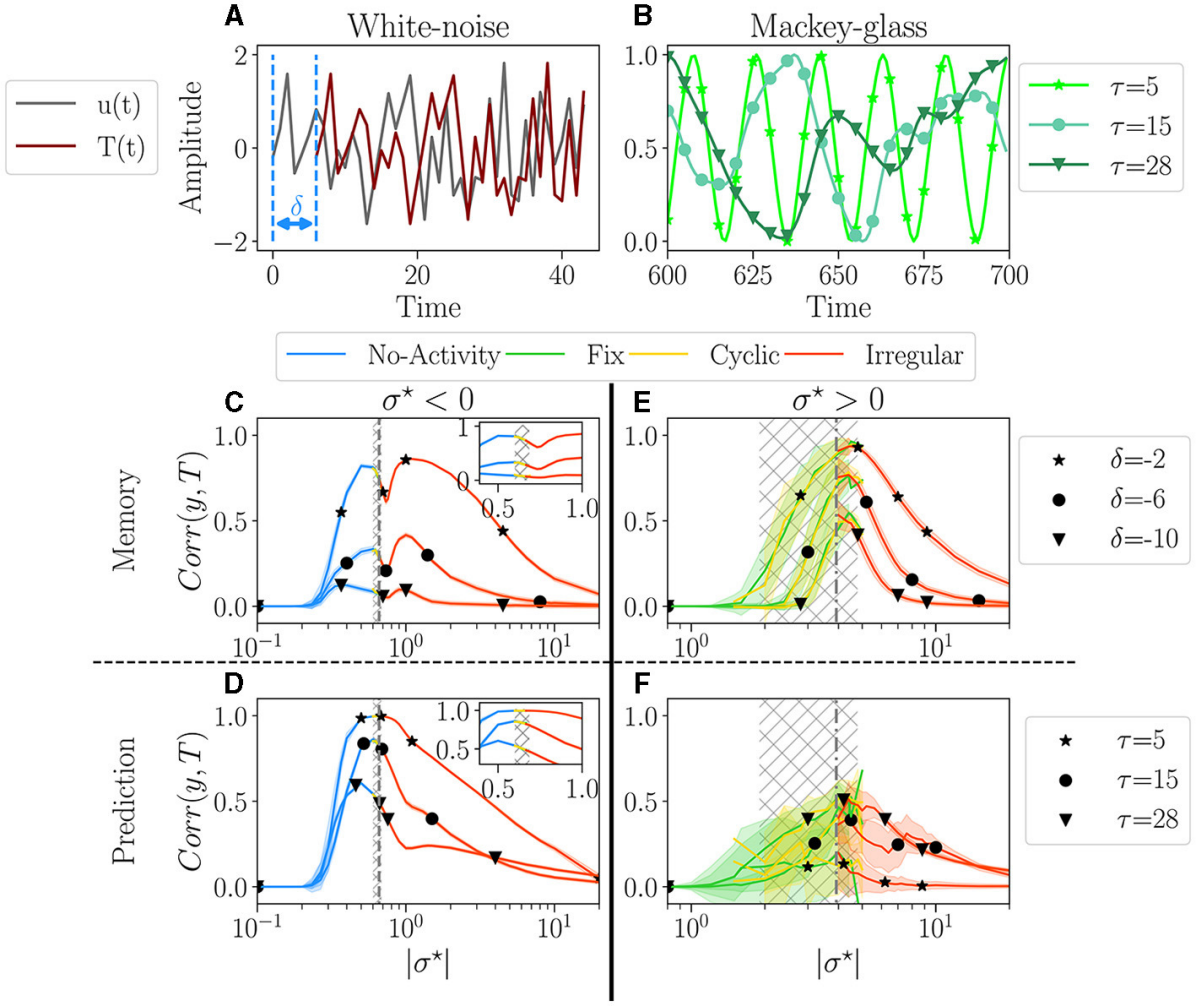
Performances for two tasks: white noise memory **(C, E)**; and Mackey-glass prediction **(D, F)**. **(A, D)** Examples of signals for each task with their respective parameters. **(A)** White noise memory task, which consists in remembering the input (gray), to reproduce it in output (dark red) with a negative delay δ (shown example corresponds to δ = −6). **(B)** Mackey glass is controlled by the parameter τ (see methodology Supplementary material S8.3 for more details), ranging from periodic to chaotic. **(C–F)** The average performance *Corr*(*y, T*) between the output *y* and target *T*, plotted over |σ^⋆^|, for each dominant attractor category *no-activity, fix, cyclic* or *irregular*. For each value of σ^⋆^ we have 100 reservoirs. The solid line then represents the average over reservoirs belonging to the same attractor category; individual reservoir performances are averaged over 5 initial conditions. The shaded area represents one standard deviation. Higher correlations indicate better performance. The hatched gray area represents the critical regions, as defined in Section 3.1. **(C, D)** The performance in the white-noise memory task; three values of δ are tested −2 (⋆), −6 (•), −10 (▾). **(E, F)** The performance of Mackey-Glass prediction (δ = +10); three values of τ are tested 5 (⋆), 20 (⋆), and 50 (▾). **(C, D)** Performance for σ^⋆^ < 0, with inside each plot a zoom on the critical region. **(E, F)** Performance for σ^⋆^> 0.

The first task consists of memorizing a purely random signal (i.e., uncorrelated white noise), and since there is absolutely no correlation in the input, only memorization is involved. Figure 5A illustrates this task for one value of δ = −6, with white noise as input *u*, and the target *T*.

For the second task, we explore the ability of the reservoir to predict a time series, δ = 10 time steps in the future. The input is the well-known Mackey-Glass time series, as it is a common benchmark of this type of task (Hajnal and Lörincz, 2006; Goudarzi et al., 2016; Canaday et al., 2018, among others), notably testing the ability to infer non-linear dynamics. The signal regularity is controlled by the parameter τ, see Figure 5B, ranging from periodic with τ = 5, to chaotic for τ = 28 (more information on the experiments in Supplementary material 8.3).

#### 4.1.2. Analysis

The performance of a reservoir is measured by computing the correlation product *Corr*(*y, T*) between the output *y* and the target *T*. A perfect match corresponds to a correlation of one, while a random output gives a zero correlation. An individual reservoir performance score is then obtained by averaging over the initial conditions. Each individual reservoir is associated with its dominant attractor, and the statistics of the performance of reservoirs are performed separately for each attractor.

#### 4.1.3. Results

The average performance is plotted as a function of |σ^⋆^| in Figures 5C, E for the memory task and in Figures 5D, F for the prediction task. The left column (Figures 5C, D) corresponds to σ^⋆^> 0, and the right column (Figures 5E, F) to σ < 0. The color of the lines corresponds to the attractor.

For σ^⋆^ < 0 (Figures 5C, D), performance increases over a very wide range of σ^⋆^, both for memory and prediction. This range includes the critical region (gray hatched area) but is vastly broader. Thus, being within the critical region is absolutely not mandatory to perform well. A shaded area in Figure 5 indicates the spreading of the results. There is none in plots Figures 5C, D, meaning that all reservoirs perform exactly the same for a given σ^⋆^. Moreover, as σ^⋆^ is increased through the critical region, the dominant attractors change [see zooms in plot Figures 5C, D], but surprisingly, there is no discontinuity in the performance. Indeed, inside the gray area, even though the four attractor categories are present, their respective performance all align. This strongly suggests that the dynamics of the reservoir, as measured in the absence of input, is irrelevant for the performance. Only the value of σ^⋆^ matters. In both tasks, the average performance decreases monotonically with increasing difficulty via τ and δ. In the memory task, we register a dip in performance close to the critical point. This goes against the common assumption that the edge of chaos is optimal for memory (Natschläger et al., 2005). In the prediction task, the peak of performance roughly coincides with the critical region, except for the greater difficulty, where the peak is slightly on the left.

The picture is very different for σ^⋆^> 0 (Figures 5E, F). First, the region in which some level of performance is observed is comparable to the critical region observed in free-running reservoirs. Second, there is substantial variability in performance across different reservoirs, as indicated by the large shaded areas. Despite this variability, there is an overall dependence of performance on σ^⋆^. The average performance of distinct dominant attractor categories is much noisier. However, despite being more noisy, the average performance of the distinct attractor categories aligns again, so there is still no evidence that the attractor category has any significant impact on the reservoir's performance. We observe that performance decreases as the difficulty of the memorization task increases, but interestingly, this trend appears to be inverted in the prediction task.

Once again, the two signs of σ^⋆^ give rise to different behavior. In particular, networks with σ^⋆^> 0 memorize better and are less reliable than those with σ^⋆^ < 0 but have poorer prediction capability. Yet, in all cases, attractors do not seem to be correlated to performance, as the top performance can be found in any of the four attractor categories.

#### 4.1.4. Discussion

Our results somewhat challenge the common assumption that the edge of chaos is optimal for performance and suggest that this is true for reservoirs with a majority of excitation but not necessarily with a majority of inhibition. Reservoirs with negative σ^⋆^ exhibit very reliable performances with very low reservoir-to-reservoir variability over a range in σ^⋆^ much broader than the critical region. Since reservoirs behave the same, in practice, it is sufficient to generate one, with σ^⋆^ at the left of the critical region. However, if the goal is optimal memorization, it is wiser to choose σ~0.4 in the critical region and try different reservoirs until finding a good one, which requires training and testing.

### 4.2. Cross-task performance

Beyond studying the performance in memorization and prediction separately, as often done (Bertschinger and Natschläger, 2004; Büsing et al., 2010), here we aim at answering the following question: are the reservoirs intrinsically good or bad, or does it depend on the task? In other words, are there general-purpose reservoirs and specialized ones?

#### 4.2.1. Analysis

We analyze the absolute value of the performance of all reservoirs independently of the control parameter. For each reservoir, we study its performance in the memory task as a function of its performance in the prediction tasks (see Section 4.1 *methodology*). For this, we fixed values of δ (memory) and τ (prediction), and we chose three levels of difficulty: simple (τ = 5, δ = −2), average (τ = 20, δ = −6) and difficult (τ = 28, δ = −10).

#### 4.2.2. Results

**Figure 6:**
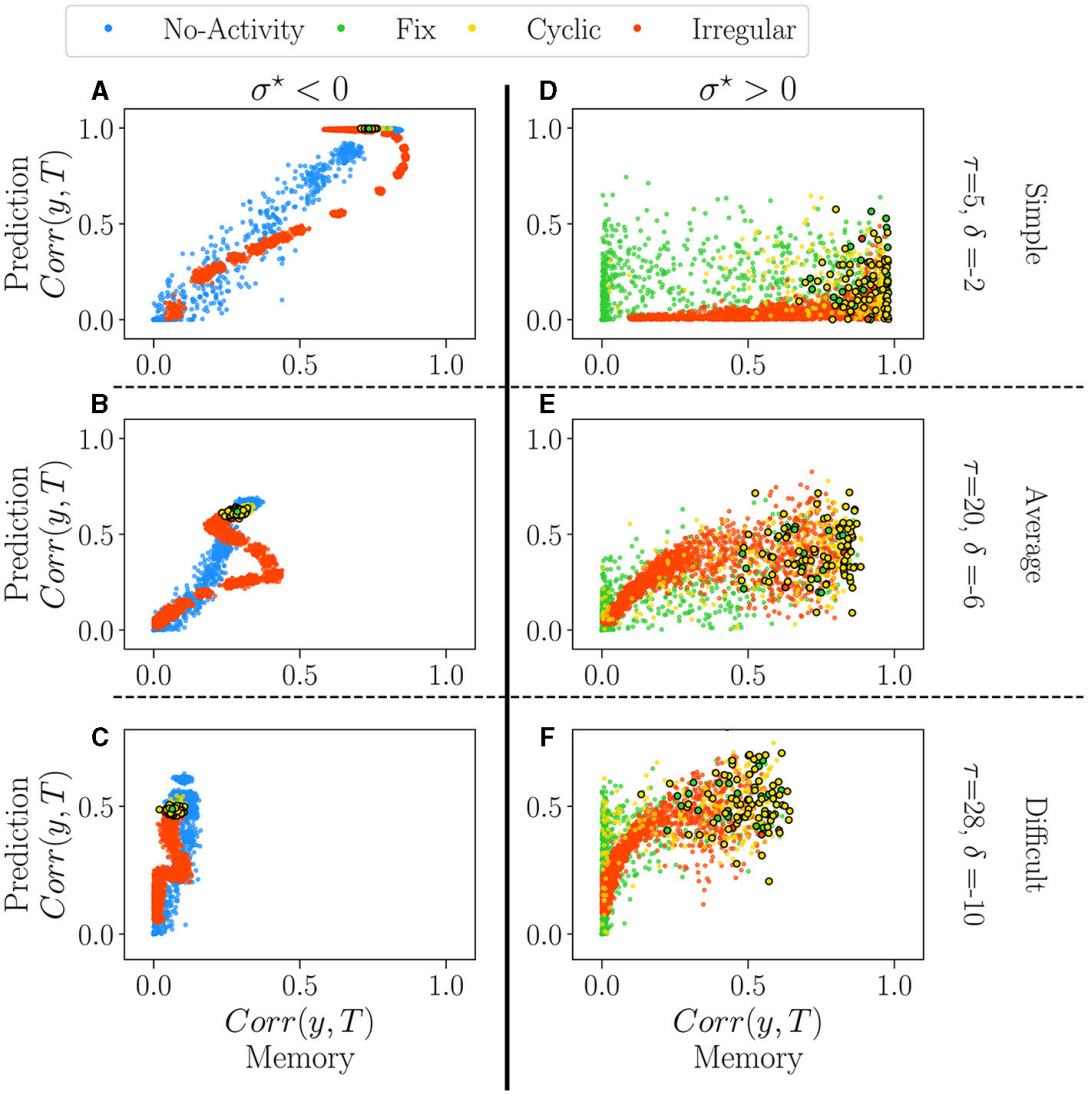
The performance of all reservoirs in the prediction tasks (Mackey-Glass) as a function of their performance in the memory tasks (white-noise), for σ^⋆^ < 0 **(A–C)** and σ^⋆^> 0 **(D–F)**. Reservoirs are again classified according to their dominant attractor (see Supplementary material S13). Each dot represents an individual reservoir performance averaged over 5 initial conditions. In all plots, dots with black edges display reservoirs taken at the critical regimes. We chose three different pairs of values for the parameters of the tasks, τ and δ, each representing a different difficulty level: 1. Simple **(A, D)** the lowest difficulty in both tasks, τ = 5 and δ = −2; 2. Average **(B, E)** average difficulty for intermediary values of τ = 20 and δ = −6; 3. Difficult **(C, F)** difficult task for higher values of τ = 28 and δ = −10.

Figure 6 displays the performance of reservoirs as colored dots. As in the previous section, each color corresponds to the dominant attractor of the reservoir. The vertical axis represents the performance in the prediction task (Mackey-Glass), and the horizontal axis that of the memory task (white noise). The columns correspond to the sign of σ^⋆^, negative on the left, positive on the right. The rows correspond to the degree of difficulty, from simple (top) to difficult (bottom).

For σ^⋆^ < 0 (left column), there is an apparent relationship in performance between the two tasks. The degree of difficulty roughly acts as a scaling factor on the curves, but the correlation is always clear. The reservoirs which are good at predicting do memorize well as well. There is, however, a reentrant region of irregular reservoirs (red) which are the best at memorizing but are not optimal for predicting, in particular for the intermediate degree of difficulty (see the red loop on Figure 6B). This point corresponds to the maximum on the right of the dip observed in Figure 5C. Interestingly, in all difficulties, reservoirs at the critical point (encircled dots) create a narrow area with a good overall performance. This picture refines our previous analysis of the impact of attractors on performance, as it seems that extinguished reservoirs can perform slightly better at memory than the others, and the same for the irregular reservoirs at prediction. Nonetheless, the difference between attractors is rather small, and it could be argued that is insignificant.

The picture is very different for σ^⋆^> 0 (right column). Performances are distributed as clouds of points. In Figure 5, we observe a large reservoir variability in both tasks. Some reservoirs are suitable for one task and bad for the other one. For intermediate and difficult tasks, some reservoirs outperform the best ones with σ^⋆^ < 0 in both memorization and prediction. Reservoirs at the critical point are found on the right of the plots, i.e. they promise good memorization but are nonetheless widely spread, especially in prediction (vertical axis). Overall, the distinct attractors occupy the space in overlapping and indistinguishable clouds; this confirms the previous analysis that attractors do not play a role in performance.

#### 4.2.3. Discussion

Correlations in performances are very different for both signs of σ^⋆^. Choosing a reservoir with σ^⋆^~−0.66 ensures a good, general-purpose reservoir but with suboptimal performance. In contrast, going into the positive side of σ^⋆^ may lead to the best reservoirs in a given task or even better general purpose reservoirs, but this comes at a price: those gems cannot be found by the statistical analysis we have performed on their free running activity.

## 5. Conclusion

One of the main issues in the field of RC is the lack of principled methodology (Rodan and Tino, 2011) for reservoir design. This article aimed to quantify the impact of the random weight generation process to better understand the relationship between connectivity, dynamics, and performance. We demonstrated that the only control parameter is the ratio σ^⋆^ = σ/μ through a Gaussian weight distribution, which indirectly regulates the excitatory/inhibitory balance. We found two critical points and observed that reservoirs typically possess a dominant attractor, regardless of their initial states.

We investigated the relationship between the performance, the control parameter, and the preferred attractor in memory and prediction tasks. Our results reveal that σ^⋆^, hence the excitatory-inhibitory balance *b*, has a strong impact on performance in the two considered tasks while the attractor dynamics have none. We showed how to select a control parameter region that ensures good performance, thus providing a very efficient way to obtain high-performance reservoirs. This region corresponds to high attractor diversity. For σ^⋆^ < 0, the critical region is narrower and does not necessarily coincide with the top of performance, while for σ^⋆^> 0, the critical region corresponds to the performing region.

For the tasks, we showed that negative σ^⋆^ values produced superior results in prediction, with reliable performance and low reservoir-to-reservoir variability. Therefore, it is sufficient to perform free-running and pick a single value of σ^⋆^, preferably close to the critical point $\sigma_{c}^{⋆}$. In contrast, positive σ^⋆^ values were found to have higher performance in memory tasks but with greater volatility. Since a given σ^⋆^ value can lead to diverse performance outcomes, generating random reservoirs and testing them during training to select the best performers is still necessary. Given enough trials, however, our findings suggest that σ^⋆^> 0 can generate the bests general-purpose reservoirs.

## 6. Future work

We tested the impact of dynamics on performance in two types of tasks: memory and prediction, for various time series. It would be interesting to test if and how the balance and attractor dynamic impact other types of tasks and inputs, notably classification, as it is also a standard task in machine learning. Moreover, extending the cross-task performance analysis to classification could potentially reveal interesting insights about its performance.

Still, one surprising result is the limited impact of the intrinsic attractor dynamics on performance. One could test the robustness of this result by refining the attractor category, and future work may reveal greater performance sensitivity to attractor dynamics. For example, the extinguished category included all reservoirs with activities dying before 1, 000 time steps. Refining the analysis could involve correlating performance with the average time before free-running reservoir activity dies out. Similarly, cyclic reservoirs could be refined by analyzing their period (Kinoshita et al., 2009), while some irregular activities may be considered cyclic when run for more extended periods. Moreover, it is possible that combining other types of analysis, such as correlation in space and time (Metzner and Krauss, 2022), avalanche distribution size (Siddiqui et al., 2018), basins of attraction (Del Giudice et al., 1998; Chinarov and Menzinger, 2000; Kinoshita et al., 2009), the number of attractors (Cabessa and Villa, 2018), and study of the reservoir topology (Kinoshita et al., 2009; Masulli and Villa, 2015), could provide better categorization of dynamics, with ultimately better predictive power of performance.

Finally, it would be of particular interest to see if our finding regarding the impact of the excitatory/inhibitory balance and dominant attractors also applies to other models, such as the quantum Ising spins, also used in the context of RC, which exhibit analogous phase transitions (Martínez-Peña et al., 2021), and improved memory and prediction of time series in its vicinity (Kutvonen et al., 2020).

## Data availability statement

The original contributions presented in the study are publicly available. This data can be found here (Calvet, 2023): https://doi.org/10.5281/zenodo.8121795.

## Author contributions

EC conducted the research under the supervision of JR and BR. EC created the model, collected and analyzed the data, and wrote the manuscript. JR provided invaluable guidance and perspective throughout the process, along with BR, who also contributed to the writing and revision of the manuscript. All authors read and approved the final version of the article.
