# Supplementary material for: Excitatory/inhibitory balance emerges as a key factor for RBN performance, overriding attractor dynamics
Source: Front Comput Neurosci. 2023 Aug 9;17:1223258. doi: 10.3389/fncom.2023.1223258 (PMC10445160; doi:10.3389/fncom.2023.1223258)
Supplement: Supplementary file 1 [file Presentation_1.pdf]

Supplementary Material

7 THE EXPERIMENTS

This article proposes two sets of experiments: in the first setting, one is interested in the free time evolution of the reservoir without input ($u_i = 0$). The second set is devoted to the measurement of the capacity of the reservoir to execute two tasks.

7.1 Free running network

Both experiments in this section are based on the same simulation, and do not require the input and readout layers:

1. At time $t = 0$ the network state is randomly initialized with provided *seed*, and a fixed 20% of active nodes ($x_i = 1$).
2. Then the network is let run for a duration $D = 2000$ time steps.

For each value of $\sigma^\star$, we generate 100 reservoirs, i.e. reservoirs having all the same architecture but different weights. We consider two ways to perform statistics.

Statistics over many reservoirs (section 3.1).

Each **reservoir** is run once in a *free run simulation*. We report the result of 90 values of $\sigma^\star < 0$, 110 values of $\sigma^\star > 0$, and 80 for $\mu = 0$, for a total of $28,000$ simulations.

Statistics of reservoirs (section 3.2).

Each **reservoir** is run 100 times with different initial conditions. We report the result of 80 values of $\sigma^\star$ for each sign, for a total of $1,600,000$ simulations.

8 THE CONTROL PARAMETER

8.1 Link between $\sigma^\star$ and ρ

The spectral radius ρ of a square matrix W is the largest of its eigenvalues in absolute value. We will consider the case where the size N of the network, here $N = 10,000$ is large enough so that ρ is self-averaging: it does not depend on the exact coefficients of W but simply on the parameters (μ, σ) of their distribution. It is showcased in Fig. 7 where the (inexistent) error bars represent the spread in the results for the calculation of ρ for different reservoirs of identical (μ, σ). One can thus consider ρ to be a function of μ and σ.

The property $\rho(\lambda W) = |\lambda|\rho(W)$ for any real λ leads to $\rho(\lambda\mu, \lambda\sigma) = |\lambda|\rho(\mu, \sigma)$. In the case $\mu = 0$, taking $\lambda = 1/\sigma$ gives $\rho(0, \sigma) = \alpha|\sigma|$ with $\alpha = \rho(0, 1)$ a parameter independent of the weight matrix. We thus find that while the dynamics of the network is independent of σ for $\mu = 0$ (see Fig. 3), ρ can take any value. In this case, there is no link between ρ and the dynamics of the network.

For $\mu \neq 0$, taking $\lambda = 1/\mu$ gives $\rho(\mu, \sigma) = |\mu|\beta(\sigma^\star)$ with $\beta(s) = \rho(1, s)$. Thus for a given value of $\sigma^\star$, ρ can take any value. There is however a direct link between β and $\sigma^\star$, shown in Fig. 7. For $\sigma = 0$, the weight matrix is $W = \mu A$ with A a matrix with K ones and $N - K$ zeroes in each row, thus obeying $\rho(A) = \beta(0) = K$, leading to $\rho(\mu, 0) = |\mu|K$, in agreement with our numerical simulation: ρ saturates at

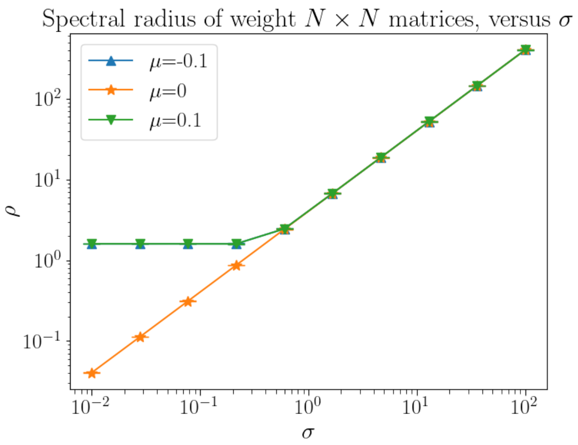

Fig. 7. The spectral radius ρ, of Gaussian weight matrices, computed for different values of the mean $\mu(W)$, and as a function of the standard deviation $\sigma(W)$. Each dot represents the average computed on 10 matrices, and errorbars represent two standard deviations.

low σ. For the limit of large σ taking $\lambda = 1/\sigma$ gives $\rho(\mu, \sigma) = \sigma\rho(1/\sigma^\star, 1) \simeq \sigma\rho(0, 1) = \alpha\sigma$, which is the result for $\mu = 0$, as observed in Fig. 7.

While it may be tempting to replace ρ by its renormalized value β, both quantities suffer from the same weakness: they do not depend on the sign of $\sigma^\star$, whereas the dynamics of the network strongly do.

8.2 Control parameter values

Below are the parameter values for the experiment of free running (see methodology in **S**7.1):

Start	End	Step
0.01	0.06	0.001
0.06	0.1	0.0007
0.07	0.1	0.001
0.1	0.2	0.01
0.2	1	0.1
1	11	1

Table 1. Values of $\sigma(W)$ for the first experiment, $\mu = -0.1$.

Start	End	Step
0.01	0.07	0.001
0.07	0.2	0.01
0.2	0.5	0.02
0.5	1	0.1
1	11	1

Table 2. Values of $\sigma(W)$ for the first experiment, $\mu = 0.1$.

8.3 Performance of tasks

All experiments in this section test the performance in a task, where learning is required for the readout layer. For all tasks, the following framework is applied. Assuming that F is the reservoir, the relationship between the input time series $u(t)$ and the output layer y is $y(t) = F(u(t))$. The goal here is to learn the target $T(t)$, such that $y(t) = T(t)$ and the target is set to:

$$T(t) = u(t + \delta) \quad (8)$$

Here, δ is an integer which represents a number of time steps. This parameter serves the general purpose of setting the type of task:

- **Memory task** for $\delta < 0$, the reservoir output must reproduce the input received in the past.
- **Prediction task** for $\delta > 0$, the network output must produce an input not yet seen by the reservoir.

The (integer) parameter δ also sets the difficulty of the task: the higher in absolute value, the more demanding it is.

White noise memory (section 4). $u(t)$ is a zero mean, unit variance white i.i.d noise. Successive inputs are uncorrelated, so prediction is not involved, only memory. We report results for $\delta \in \{-14, -10, -6, -2\}$ (see figure 4.**A** for an illustration).

Prediction of Mackey-Glass series (section 4). $u(t)$ is the Mackey-Glass time series (Hajnal und Lörincz, 2006), which is common to benchmark reservoir computational capabilities. It is given by the following dynamical equation:

$$x_{t+1} = ax_t + \frac{bx_{t-\tau}}{c + x_{t-\tau}^d} \quad (9)$$

We choose $a = 0.9$, $b = 0.2$, $c = 0.9$, $d = 10$ and $x_0 = 0.1$. The dynamic of this equation can be controlled by varying τ: as τ increases, the time series evolves from periodic ($\tau = 5$) to chaotic ($\tau = 28$), with a continuous increase in complexity in between. Results are for a fixed $\delta = 10$ and $\tau = \{5, 15, 20, 28\}$ (see figure 4.**B** for an illustration).

8.4 Training

The two tasks follow the same protocol for training the readout:

- The network receives the input u for duration $D = 2000$ time steps.
- The first 500 time steps are discarded and considered transients. This value is empirically obtained, adapted from the signal at hand, and coupled to a convergence test for permanent regime detection.
- The training is performed on the following 1500 time steps on the concatenated in-time reservoir outputs, using the optimization procedure described in 2.3.

Each experiment consists in 40 values of $\sigma^\star$, 100 reservoirs per $\sigma^\star$ value, and each network is run 5 times with different randomly tossed inputs (i.e., $40,000$ simulations). Each training is performed for 4000 epochs (with a total of $640,000,000$ training epochs).

8.5 Performance

The metrics of performance are given by the *Pearson correlation coefficient* between the output and the target (each of length 1500). A perfect match corresponds to a correlation of 1 while 0 means an output of the network is not better than random.

9 MEASURE OF NETWORKS DYNAMICS

To evaluate the reservoir dynamics, we used the most straightforward measure one can imagine: the *activity*, which we defined as the sum of all spikes at each given time step, see supplementary material 10. We

will take some time to make the reader appreciate the usefulness of such an approach. The sum of binary spikes is mathematically equivalent to how the magnetic field of spins in the ISING models (Ising, 1925) is computed. In ISING, the electron spins are modelled as miniature magnets that can take binary values $[-1, 1]$), which flip with some probability depending on temperature and the coupling with other spins. The ISING model is one of the closest physical models to neural networks. In fact, it has been widely used to design neural networks, for example, in the famous Hopfield network (Hopfield, 1982) or the Boltzmann machine (Sherrington und Kirkpatrick, 1975). So one could argue that our activity signal is close to what Magnetoencephalography (MEG) is for brain activity (Hämäläinen u. a., 1993).

Since this analysis does not depend on the micro-constituents, we make the case that the methodology performed in this article is easily transferable to other neural models and to other fields, such as neurosciences, physics, and the study of complex dynamical systems.

Activity: we define $A(t)$, the averaged activity of the network at time t, and for simplicity we will refer to it as *activity*. It is the normalized sum of all neural states $x_i(t)$:

$$A(t) = \frac{1}{N} \sum_{i=1}^{N} x_i(t)$$

Steady state of reservoir: In the main text (part 3.1), we defined the steady state as A for ease of notation, here for the sake of clarity, we define the steady activity A_s of free-running reservoirs during $D = 2000$ time steps, as $A_s = A(t \geq D/2)$, the activity of the last $D - D/2 = 1000$ time steps of free running simulations.

10 METRICS

10.1 Permanent regime statistics:

here are two statistical analyses we perform on the measure of the steady state activity A_s of a given reservoir, where $\overline{(.)}$ represents the time-average over a quantity, and $\delta A(t) = A(t) - \bar{A}$:

- Permanent regime time-average:

$$\bar{A}_s = \frac{2}{D} \sum_{t=D/2}^{D} A(t)$$

- Permanent regime time-variance:

$$\overline{\delta {A_s}^2} = \frac{2}{D} \sum_{t=D/2}^{D} (A(t) - \bar{A}_s)^2$$

Average over reservoirs of permanent regime statistics: in the *first experiment* (see results in section 3.1), for each value $\sigma^\star$, we compute the average (over reservoirs generated with 100 different seeds) of the permanent regime *average* and *variance* (overtime time step t). Here A_s^r denotes the stationary activity of the reservoir of index r generated with $seed = r \times 100$, for a total number of reservoir $R = 100$. We compute the average over reservoirs of the permanent regime statistics, where the back $\langle . \rangle$ denotes the average over reservoirs:

- Average over reservoirs of the permanent regime average:

$$\langle \bar{A_s} \rangle = \frac{1}{R}\sum_{r=1}^{R} \overline{A_s^r}$$

- Average over reservoirs of the permanent regime variance:

$$\langle \overline{\delta A_s{}^2} \rangle = \frac{1}{R}\sum_{r=1}^{R} \overline{(\delta A_s^r)^2}$$

Statistics of the BiEntropy: the BiEntropy solves one important limitation of the Shannon entropy, regarding the evaluation of binary words: as an example, let us take these two binary words ”01010101” and ”10110010”. Although one is fully periodic and the other is somewhat random, they both have the same probability of occurrence of 1’s and 0’s; hence they have the same Shannon entropy. The Shannon entropy is thus insufficient to inform on the regularity versus the disorder of binary words. In contradiction, the binary entropy has been designed to discriminate patterns that the Shanon entropy could not (Blackledge und Mosola, 2020). We argue that in our context this metric is perfectly fitted to evaluate the binary words of spike time patterns. Since phase transition in such systems are known to be between an ordered and disordered phase, while the critical regime, also known as the edge of chaos, is supposedly a mixture of both. One property of particular interest to us regarding that metric, is that it is bounded between between 0 (for perfectly ordered words), and 1 (totally disordered).*

- BiEntropy of permanent regime: the BiEntropy H_b is computed on binarized steady activity δA_s. First, we subtract the mean $A_s - \bar{A}_s$, and then all positive values are clamped to one and negative values to zero, resulting in a binary sequence. The H_b is then computed on this sequence after converting it to a string.
- Average over reservoirs of the permanent regime BiEntropy:

$$\langle H_b \rangle = \frac{1}{R}\sum_{r=1}^{R} H_b^r$$

- Variance over reservoirs of the permanent regime BiEntropy:

$$\langle \delta H_b{}^2 \rangle = \frac{1}{R}\sum_{r=1}^{R} (H_b^r - \langle H_b \rangle)^2$$

NB: given that neuron states are binary, one could wonder why employing a binarized version of a continuous variable like A. The reason is twofold: 1. the number of neurons in the network is $N = 10000$. Therefore, computing H_b on all neurons would be extremely costly. As such, reducing the number of neurons would be crucial, though it would necessitate a criterion for selection. This poses the risk of missing important information or introducing biases. 2. This would not be applicable in many real-world applications where access to the micro-constituents of the reservoir is difficult or even impossible. As such, we ensure our methodology is easily transferable to other areas comprising non-invasive studies of the brain.

* For example, the word ”01010101” has $H_b = 0.0078$, and the word ”10110010” has $H_b = 0.7596$. A convenient way to discriminate both words.

11 CLASSIFICATION OF ATTRACTORS

In this section, we provide a more grounded explanation of the choice of attractor categories. In the main text (part 3.2), we developed a scheme to categorize activities by their respective attractor. Fig. 4 showed the histograms of attractors as a function of $\sigma^{\star}$, and in this section, we provide a refined version of these statistics by adding two more categories of attractor:

- *Saturated attractors*: The steady activity is saturated when all neurons are active at all times.
- *Non-trivial*: Any signal whose category of dynamics changes over time and that does not fit in previously mentioned types is considered non-trivial. This comprises cases where during the time window considered, activity suddenly changes from one type of dynamic to another.

We show in Fig. 8 examples of the various activities belonging to each category, No-Activity and *Fix* (**A**), *Cyclic* (**B**), *Irregular* (**C**), and lastly, *Non-Trivial* (**D**). In the following paragraphs, we explain in more detail some specificities of the *fixed* point attractor category and the reason why did not treat the *Non-trivial* case separately from the irregular ones. First, we must mention that the *fixed* point attractor category could, in theory, encompass the *extinguished* and *saturated* cases as well. This is because they all fit inside the definition of a time derivative of zero. The reason why we separated inactive reservoirs from the two others comes from percolation theory (Coniglio u. a., 1976): formally, a reservoir has not percolated if the activity does not spread to infinity in space and/or time. While at the percolation threshold, activity will start to propagate indefinitely. We make the case that the percolation threshold, which does not coincide here with the critical points, constitutes another type of transition (Cohen u. a., 2010), from inactive, to active, hence the distinction. This is visible in Fig. 8.**E**, as the fixed point attractors appear a bit before the *cyclic* ones, around $\sigma^{\star} \sim 0.5$.

Second, in Fig. 8.**F**, we can see that the transition from *Saturated* to *Fix* attractor happens very early (in terms of $\sigma^{\star}$), compared to the rest of the attractors. So early, in fact, that it is not part of the phase transition. As you might recall, the analysis performed in part 3.1 revealed that this region has a zero variance of both activity and BiEntropy. We conclude that the transition from *Saturated* to *Fix* is not related to a change of dynamics but only to a change in amplitude. As a result, we have chosen not to differentiate the two.

Thirdly, as one can clearly see in both Fig. 8.**E** and **F**, the *Non-trivial* dynamics are very rare. As such, it is worth mentioning that in the analysis performed in **S**13, where we categorize reservoirs depending on their dominant attractors, not a single reservoir exhibits a dominance of *Non-Trivial* dynamics. This is important because it means this category of attractors is irrelevant for finding correlations between dominant attractors and performance.

12 DIVERSITY OF RESERVOIR ATTRACTOR DISTRIBUTIONS

We compute the Shanon entropy H_s, with the goal of quantifying how varied are the attractor distributions of given reservoirs. Typically, if a reservoir activity always falls into one attractor, irrespective of the initial state, the Shanon entropy will give 0. On the other hand, the maximum entropy is obtained for a uniform distribution where each attractor is obtained $1/4$ of the time, and $H_s^{max} = -log(1/4)$. The Shanon Entropy is therefore normalized by its maximum value and averaged over the 100 reservoir of each value of $\sigma^{\star}$.

To quantify reservoir dynamics diversity, we plotted the average normalized entropy of reservoir attractor distributions, $\langle H_s/H_s^{max} \rangle$, against the control parameter for $\sigma^{\star} < 0$ (**A**) and $\sigma^{\star} > 0$ (**B**). The shaded area represents one standard deviation. Lower values of this quantity indicate a stronger dominance of one

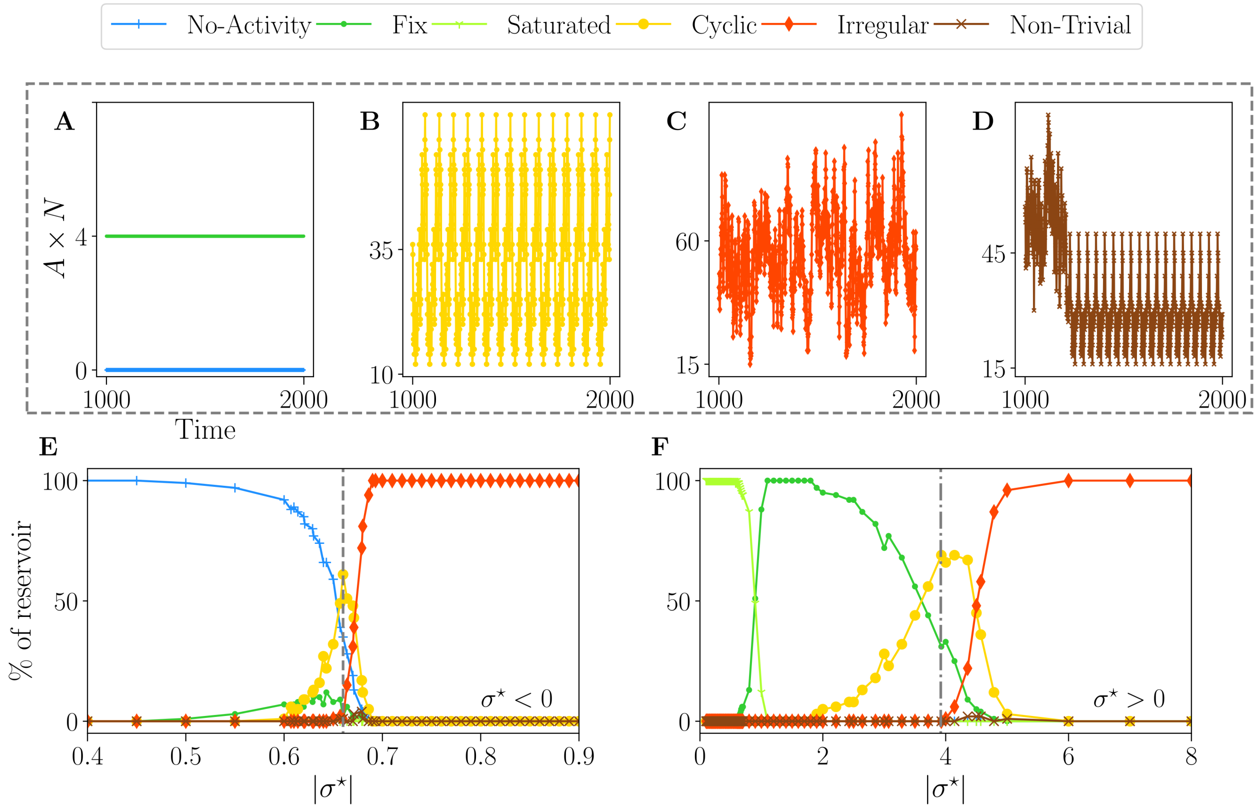

Fig. 8. Examples of activities, classified into their respective category of attractor: (**A**) no-activity (+), fix (•); (**B**) cyclic (•), (**C**) irregular (♦), and (**D**) non-trivial (×). The percentage of trials (or activities) belonging to each category of attractors is plotted against the control parameter $\sigma^\star$. (**A**) the phase transition for $\sigma^\star < 0$, and (**B**) the phase transition obtained with $\sigma^\star > 0$. In both cases, we zoom on the critical regions since the ordered, and irregular regimes have constant statistics. **E** and **F**: the statistics of attractors of free running simulations are computed for each value of $\sigma^\star$, for which 100 reservoirs are run once (see methodology 7.1). (**E**) $\sigma^\star < 0$, and (**F**) $\sigma^\star > 0$.

attractor. Outside of the critical region, initial conditions are irrelevant, and reservoirs always converge to a single attractor. The region where $\langle H_s \rangle$ increases correspond precisely to the region where the BiEntropy, shown in Fig. 9, is nonzero. In this region, different reservoirs may lead to different attractors. There is significantly more entropy, i.e. more competition between attractors, for $\sigma^\star < 0$.

The higher variance in H_s also implies increased reservoir-to-reservoir variability. This variability is more pronounced for $\sigma^\star < 0$, with the average entropy exceeding 0.25 near the critical point and the shaded area approaching 0.5. For $\sigma^\star > 0$, reservoirs generally exhibit stronger dominant attractors, as the standard deviation never surpasses 0.25. Despite these differences, the normalized entropy remains consistently below 0.5 for all values of $\sigma^\star$, irrespective of the sign. This value approximately corresponds to an attractor distribution where one attractor dominates 80% of the initial conditions, indicating that most reservoirs possess a peaked distribution with a predominant attractor.

13 STATISTIC OF DOMINANT ATTRACTORS

Fig. 10 displays the statistics of the dominant attractors of each of the 100 reservoirs generated by $\sigma^\star$ values. As one can note, the statistics are also unchanged from Fig. 4. Taken together, these results indicate strong statistical robustness, as averaging over reservoirs is almost equivalent to averaging reservoirs themselves.

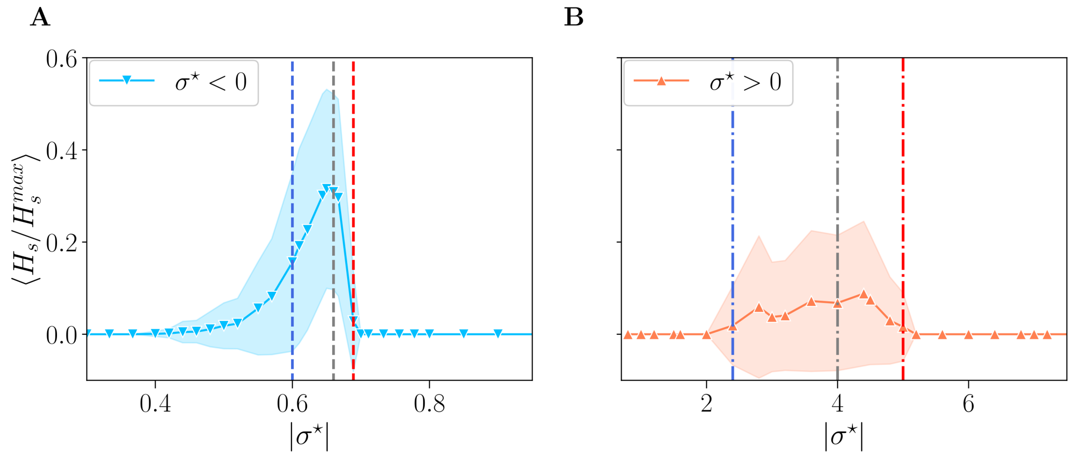

FIG. S3 – The normalized Shanon entropy of individual reservoir attractor distributions. **A** and **B**: The average over reservoirs entropy of the entropy H_s versus the control parameter $|\sigma^\star|$, for $\sigma^\star < 0$ (**A**) and $\sigma^\star > 0$ (**B**). The shaded area represents one standard deviation. The coloured hashed lines correspond to the boxes of Fig. 4: (blue) $\sigma^\star = -0.6$ (**A**) and $\sigma^\star = 2.4$ (**D**), (gray) $\sigma^\star = -0.66$ (**B**) and $\sigma^\star = 4.0$ (**E**), (red) $\sigma^\star = -0.689$ (**C**) and $\sigma^\star = 5.0$ (**F**).

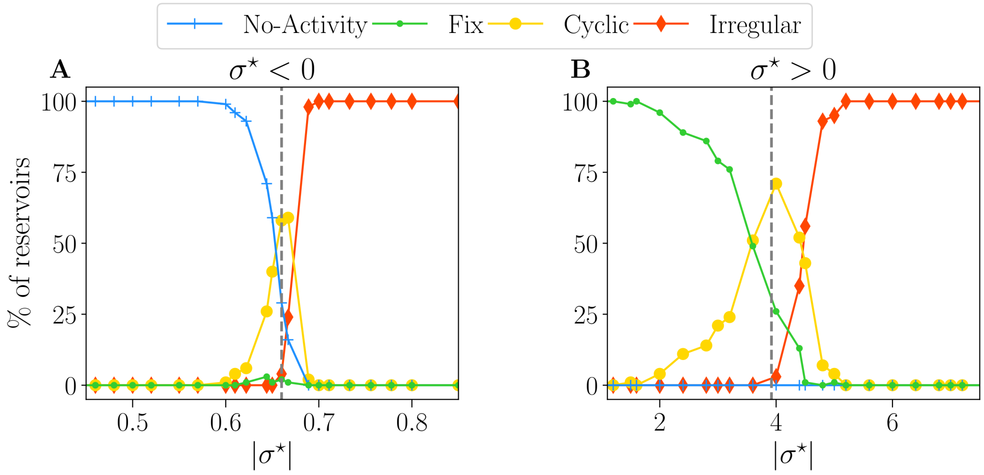

FIG. S4 – The statistics of reservoir dominant attractors. For each value of $\sigma^\star$, 100 reservoirs are run 100 times with different initial conditions (see **S**1.1). The resulting activities are classified into the category of attractors: no-activity (+), fix (•), cyclic (•), and irregular (♦). The statistics of reservoirs with specified dominant attractor is plotted against the control parameter $\sigma^\star$. (**A**) $\sigma^\star < 0$, (**B**) $\sigma^\star > 0$.

REFERENCES

[Blackledge und Mosola 2020] BLACKLEDGE, J. M. ; MOSOLA, N.: A Statistically Significant Test to Evaluate the Order or Disorder of a Binary String. In: *2020 31st Irish Signals and Systems Conference (ISSC)*, IEEE, 6 2020, S. 1–6. – URL `https://ieeexplore.ieee.org/document/9180178/`. – ISBN 978-1-7281-9418-9

[Cohen u. a. 2010] COHEN, O. ; KESELMAN, A. ; MOSES, E. ; RODRÍGUEZ MARTÍNEZ, M. ; SORIANO, J. ; TLUSTY, T.: Quorum percolation in living neural networks. In: *EPL (Europhysics Letters)* 89 (2010), 1, Nr. 1, S. 18008. – URL `https://iopscience.iop.org/article/10.1209/0295-5075/89/18008`. – ISSN 0295-5075

[Coniglio u. a. 1976] CONIGLIO, Antonio ; NAPPI, Chiara R. ; PERUGGI, Fulvio ; RUSSO, Lucio: Percolation and phase transitions in the Ising model. In: *Communications in Mathematical Physics* 51

(1976), 10, Nr. 3, S. 315–323. – URL http://link.springer.com/10.1007/BF01617925. – ISSN 0010-3616

[Hajnal und Lörincz 2006] HAJNAL, Márton A. ; LÖRINCZ, András: Critical echo state networks. In: *Lecture Notes in Computer Science (including subseries Lecture Notes in Artificial Intelligence and Lecture Notes in Bioinformatics)* 4131 LNCS (2006), Nr. September 2006, S. 658–667. – ISBN 3540386254

[Hämäläinen u. a. 1993] HÄMÄLÄINEN, Matti ; HARI, Riitta ; ILMONIEMI, Risto J. ; KNUUTILA, Jukka ; LOUNASMAA, Olli V.: Magnetoencephalography theory, instrumentation, and applications to noninvasive studies of the working human brain. In: *Reviews of Modern Physics* 65 (1993), Nr. 2. – ISSN 00346861

[Hopfield 1982] HOPFIELD, J. J.: Neural networks and physical systems with emergent collective computational abilities. In: *Proceedings of the National Academy of Sciences of the United States of America* 79 (1982), Nr. 8. – ISSN 00278424

[Ising 1925] ISING, Ernst: Beitrag zur Theorie des Ferromagnetismus. In: *Zeitschrift für Physik* 31 (1925), Nr. 1. – ISSN 00443328

[Sherrington und Kirkpatrick 1975] SHERRINGTON, David ; KIRKPATRICK, Scott: Solvable model of a spin-glass. In: *Physical Review Letters* 35 (1975), Nr. 26. – ISSN 00319007
